# Evaluation of Exposure to Arsenic in Residential Soil

**DOI:** 10.1289/ehp.8178

**Published:** 2005-08-17

**Authors:** Joyce S. Tsuji, Maria D. Van Kerkhove, Rhonda S. Kaetzel, Carolyn G. Scrafford, Pamela J. Mink, Leila M. Barraj, Eric A. Crecelius, Michael Goodman

**Affiliations:** 1 Exponent, Bellevue, Washington, USA; 2 Exponent, New York, New York, USA; 3 Exponent, Washington, DC, USA; 4 Battelle Marine Sciences Laboratory, Sequim, Washington, USA; 5 Emory University, Atlanta, Georgia, USA

**Keywords:** arsenic, biomonitoring, exposure, soil, urine

## Abstract

In response to concerns regarding arsenic in soil from a pesticide manufacturing plant, we conducted a biomonitoring study on children younger than 7 years of age, the age category of children most exposed to soil. Urine samples from 77 children (47% participation rate) were analyzed for total arsenic and arsenic species related to ingestion of inorganic arsenic. Older individuals also provided urine (*n* = 362) and toenail (*n* = 67) samples. Speciated urinary arsenic levels were similar between children (geometric mean, geometric SD, and range: 4.0, 2.2, and 0.89–17.7 μg/L, respectively) and older participants (3.8, 1.9, 0.91–19.9 μg/L) and consistent with unexposed populations. Toenail samples were < 1 mg/kg. Correlations between speciated urinary arsenic and arsenic in soil (*r* = 0.137, *p* = 0.39; *n* = 41) or house dust (*r* = 0.049, *p* = 0.73; *n* = 52) were not significant for children. Similarly, questionnaire responses indicating soil exposure were not associated with increased urinary arsenic levels. Relatively low soil arsenic exposure likely precluded quantification of arsenic exposure above background.

Arsenic occurs naturally in the environment, and inorganic forms are of greatest health concern [Agency for Toxic Substances and Disease Registry (ATSDR) 2000]. Arsenic in soil has been the focus of regulatory action at sites in the United States for which health risk assessments are used to guide decisions on soil cleanup. Communication of risk assessment results, however, may lead people to believe that their cancer risk is substantial and to desire medical tests. Biomonitoring is typically offered to indicate whether exposures, and presumably risks, are above background (ATSDR 2000).

In a small U.S. community in New York State (Middleport), historical pesticide manufacture was associated with arsenic in soil [U.S. Environmental Protection Agency (U.S. EPA) 2002b]. Soil sampling and remediation initially focused on the plant site (FMC Corporation), adjacent school property, and drainage ditches and creeks that received surface water flow from the plant. Several residential properties near the plant site or along drainages also had arsenic soil levels in excess of the state cleanup level of 20 mg/kg.

Health risks from soil were of concern to the local regulatory agency and the community. At the request of community representatives, we conducted an exposure study that focused on young children for arsenic biomonitoring and primary analyses of soil arsenic exposure. Preschool-age children are considered the most exposed age group for chemicals in soil (Polissar et al. 1990; U.S. EPA 2002a).

## Materials and Methods

In the summer and fall of 2003, Middleport residents were offered sampling of urine, toe-nails, soil, house dust, and homegrown produce, but not drinking water because the community is supplied by a water district [< 5 μg/L arsenic; Niagara County Water District (NCWD) 2004; U.S. EPA 2002b].

### Study population.

Recruitment focused on young children (i.e., < 7 years of age), although residents of all ages were informed of the study and allowed to participate. In addition to community meetings, notices, and mailings, all houses in the study area were systematically visited for census and recruitment. Repeated attempts were made as needed, especially for houses with evidence of children (according to neighbors, town clerk, presence of toys, etc.). Participation required review of study information, written consent, and completion of a questionnaire on demographic, socioeconomic, and behavioral information and housing characteristics. At the time of urine collection, participants (or parents) completed a questionnaire of dietary habits, activities, conditions, and behaviors potentially related to arsenic exposure.

### Biomarkers.

#### Urine.

Participants provided two first-morning-void urine samples on consecutive days between 1 August and 13 September. Participants were asked not to eat seafood for 3 days before sampling. Participants received urine collection kits (including pediatric urine bags for nontoilet-trained children) the day before collection. After collection, urine samples were stored on frozen gel packs or refrigerated before delivery to Lockport Memorial Hospital laboratory (Lockport, NY) for measurement of creatinine analysis by colorimetric method (values for the two first-morning-void samples were averaged). Samples were then shipped on frozen gel packs by courier to Battelle Marine Sciences Laboratory (Sequim, WA) and frozen until arsenic analysis. Quality assurance procedures were followed for all phases of data collection for urine and other samples.

Battelle Marine Sciences Laboratory analyzed composite urine samples (10 mL from each daily sample) for total arsenic by inductively coupled plasma–mass spectrometry (ICP-MS) (U.S. EPA 1996a) with a method detection limit of 0.2 μg/L. After further acidification of diluted samples with hydrochloric acid to pH < 2 and reduction with sodium borohydride, arsenic species (i.e., those related to ingestion and metabolism of inorganic arsenic) were trapped on a chromatography column and analyzed with hydride generation atomic absorption spectroscopy (U.S. EPA 1996b). Target method detection limits for arsenic species—inorganic arsenic, mono-methylarsonic acid (MMA), and dimethylarsinic acid (DMA)—were 0.06, 0.4, and 0.08 μg/L, respectively, with some estimated MMA and DMA values below these limits. In statistical analyses, undetected arsenic species in urine were conservatively assigned a level of 0.25 μg/L (half the method reporting limit).

In addition to analysis of standard quality control samples, 1 in 20 samples was analyzed by the U.S. Centers for Disease Control and Prevention (CDC) Inorganic Toxicology Laboratory (Atlanta, GA) for total arsenic, inorganic arsenic, MMA, DMA, arsenobetaine (AsB), trimethylarsine oxide, and arsenocholine (detection limits of 1.2, 1.0, 0.9, 1.7, 0.4, 1.0, and 0.6 μg/L, respectively). The latter two arsenic forms were not detected. AsB, an organic arsenic form in foods such as fish, was detected by the CDC in most of the 24 split samples. The results of both laboratories were highly correlated for total arsenic (*R*^2^ = 0.99; CDC = Battelle × 1.1 − 5.58) and reasonably correlated for speciated arsenic (*R* ^2^ = 0.67; CDC = Battelle × 0.68 + 2.96), given the differences in analytical techniques and detection limits.

#### Toenails.

Participants were informed that a condition of toenail sampling was wearing shoes outdoors for a month before collection. Those requesting sampling were given collection materials. Because of the time required to collect a sufficient sample (0.5 g requested), toenail samples were received from late August through October. Samples were scored for visible dirt/discoloration from 1 (clean) to 4 (all clippings dirty/discolored). Of the 84 samples submitted, 67 (none from young children) had sufficient mass for analysis (0.05 g). Toenail samples prepared according to Karagas et al. (2000) (nail polish removed with acetone, if necessary; sonicated in deionized water for 10 min; rinsed in deionized water) were acid digested and analyzed by ICP-MS using Method 6020 (method detection limit = 0.02 mg/kg; U.S. EPA 1986).

### Environmental samples.

#### Soil.

Geomatrix Inc. (Amherst, NY) collected composite soil samples for yard, play area, and garden areas within properties using an approach similar to that of Hwang et al. (1997a). Yard soil composites included subsamples from randomly selected locations (at least 3 m apart) within each of a minimum of four representative sectors and two to six additional composite samples for yard areas > 1,000 m^2^. Low areas near drainages were sampled as a separate composite. Play area composites included a minimum of four subsamples with an additional sub-sample for every 59 m^2^ in excess of 230 m^2^. Yard and play area soil was sampled at a 0–7.6 cm (0–3 in.) depth below any vegetative cover. Vegetable garden soil was collected as separate composites at 0–15 cm (0–6 in.) and 0–30 cm (0–12 in.) depths within each vegetable garden, with additional locations sampled for every 2.3 m^2^ of area.

Soil samples and standard field control samples were analyzed by H2M Laboratories (Melville, NY) for total arsenic using trace ICP-atomic emission spectroscopy using Methods 3050B/6010B (U.S. EPA 1986), with a targeted quantitation limit of 1 mg/kg. Field and laboratory quality control samples were within standard accuracy and precision limit goals.

In addition to the 77 families who consented to soil sampling, soil data from discrete sample locations were available for eight additional properties (none with children < age 7) sampled during the site remedial investigation. Discrete samples within a property were averaged.

Soil arsenic data within properties were evaluated as arithmetic mean arsenic level of 0–6 in. depth in the garden, play area, and yard samples and maximum arsenic level among these areas.

#### House dust.

Sandler Occupational Medicine Associates Inc. (Gaithersburg, MD) sampled house dust between 3 September and 11 December. Residents were instructed not to sweep or vacuum the week before sampling. Although approximately half did not comply with this request, lack of compliance did not affect house dust results.

Based on methods of Hwang et al. (1997a) and Que Hee et al. (1985), dust samples were collected with a vacuum pump through tubing into a cassette with a 0.8 μm filter at 2.5 L/min. A composite sample (0.5 g minimum) was obtained using a 625 cm^2^ template in at least three locations: the most used entrance, most frequently occupied room (living room, kitchen, or family room), and child’s bedroom. H2M Laboratories analyzed filters for arsenic (same methods as for soil).

#### Produce.

Homegrown produce was sampled in August and early September as a service to residents, not as a comprehensive survey. Battelle Marine Sciences Laboratory freeze-dried and ball-milled samples, digested approximately 0.5 g in 2 M sodium hydroxide at 80°C for about 16 hr, and analyzed for total arsenic (ICP-MS; target method detection limit = 0.062 mg/kg, dry weight).

### Data analysis.

The outcome measure of primary interest (dependent variable) was speciated arsenic in urine (i.e., sum of inorganic arsenic, MMA, and DMA). Exposure measures of primary interest (independent variables) were soil and house dust arsenic data. Other potential sources of arsenic exposure (e.g., diet), mediators of soil exposure (e.g., mouthing behaviors), and other covariates were ascertained through the questionnaire responses. Data were analyzed using the statistical software SPSS for Windows (version 7.0; SPSS, Chicago, IL) and Microsoft Excel (Microsoft Corporation, Redmond, WA).

Variables with little variation were excluded from the inferential analyses (except for those of interest, e.g., playing in creeks), and some categories were collapsed because of sparse numbers. Environmental and bio-marker data were log transformed based on their distribution (Hwang et al. 1997a). The log-transformed distributions were not significantly different from a normal distribution, except for speciated urinary arsenic, for which log transformation improved the fit with respect to normality (change in *p*-value from 0.007 to 0.013; Kolmogorov-Smirnov test of normality).

We estimated simple bivariate Pearson correlation coefficients among the dependent variable, exposure variables of primary interest, and continuous variables derived from the questionnaires. Analysis of variance and *t*-tests were conducted, where applicable, to evaluate associations between the primary outcome and independent variables and other variables derived from the questionnaires.

Linearity of relationships was examined visually before conducting regression analyses. Age-adjusted regression models that included speciated arsenic in urine with each of the environmental variables (i.e., soil and house dust arsenic levels) were run to identify a “base” model from which to build multiple regression models (including dependent and independent variables that appeared to best characterize the exposure–outcome association). To be conservative, variables with *p* < 0.15 in the age-adjusted models were included.

To evaluate possible nonindependence of subjects within families, analyses were also conducted using one randomly selected subject per family. Because both analyses yielded similar results, all subject samples were treated as independent samples regardless of household.

## Results

### Community and participant demographics.

Of the 826 households in the study area, 39 were vacant, and 55 could not be contacted but had no evidence of children. These 55 homes (mostly apartments) were assumed to have one adult resident of unknown age (average of vacant or one or two persons).

**Figure 1:**
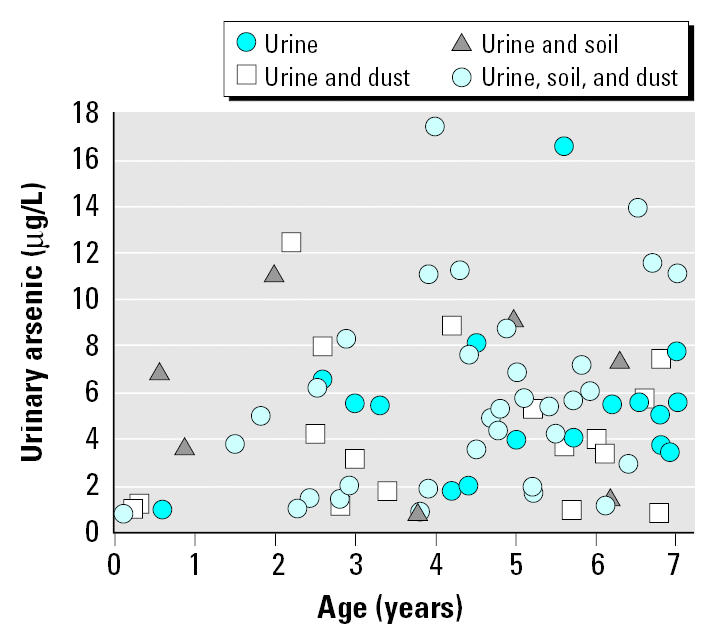
Speciated urinary arsenic levels of children younger than 7 years of age according to age. Soil and house dust sampling for individuals is noted.

Census results and the study population were generally similar to 2000 U.S. Census data (U.S. Census Bureau 2000) for Middleport (Table 1). Although the study area included outlying areas, the study area population outside Middleport was low. Forty-seven percent of children younger than 7 years of age, 48% of children younger than 13 years, and 23% of all ages of the study area population participated in urine sampling (Table 1). Soil and house dust samples were collected for 58 and 73%, respectively, of participating children younger than 7 years of age. Sampling for urinary arsenic, house dust, and soil was reasonably representative across ages (data for children shown in Figure 1). House dust and soil arsenic values were also relatively evenly distributed over age with no apparent interactions.

### Urine.

**Figure 2:**
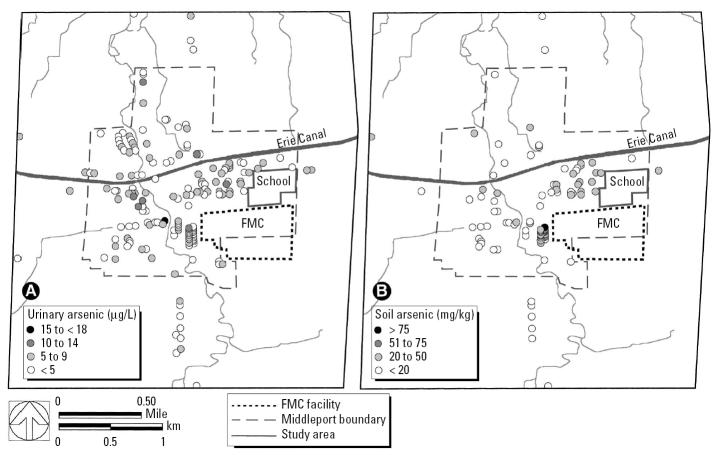
Geographic distribution of (*A*) average value of speciated arsenic in urine per family, including all participants (distribution for children is similar), and (*B*) average yard soil concentration data.

Speciated arsenic levels in the urine were < 20 μg/L and not significantly different between young children and older participants (Table 2). The geographic distribution of the speciated urine data showed little relation to the FMC Corporation facility or historical drainage from the plant (Figure 2A). By comparison, higher soil arsenic concentrations tended to be located near the plant site and along drainages to the east and north (Figure 2B).

### Toenails.

Toenail samples were < 1 mg/kg [geometric mean (GM) = 0.13 mg/kg; geometric SD (GSD) = 2.53 mg/kg; range = 0.02–0.97 mg/kg], despite evidence of surface contamination. Toenail arsenic levels increased about 75% per unit increase in discoloration score (*R*^2^ = 0.205, *p* = 0.0001) and were not correlated with speciated arsenic in urine.

### Soil and house dust.

Arsenic levels in soil averaged (GM) approximately 20 mg/kg and were < 100 mg/kg except for a few discrete samples from the properties sampled during the remedial investigation (Table 3). The highest maximum (1,124 mg/kg) and average (340 mg/kg) sample values were from the same property. The second highest maximum and average sample values were 103 mg/kg and 69 mg/kg, respectively.

Of the 111 households consenting to house dust sampling, 70 also had soil samples taken. The contribution of arsenic in soil to arsenic in house dust appears to be low and could not be quantified. Arsenic concentrations in house dust were generally lower than in soil (Table 3). Arsenic concentration or surface loading in house dust was not correlated with average or maximum soil concentration for properties with children younger than 7 years of age or for all properties sampled.

### Produce.

Twenty-five types of produce from 42 gardens had arsenic concentrations < 0.6 mg/kg (wet weight). Tomatoes, the most prevalent crop (37 gardens), had arsenic concentrations near or below the limit of detection (≤ 0.010 mg/kg). Small sample sizes of other types of vegetables and low tomato results precluded analysis of correlations of arsenic levels in vegetables with soil or biomarkers.

### Biomarker and environmental arsenic correlations.

Speciated arsenic in urine was not correlated with arsenic in soil or house dust for children younger than 7 years of age (Table 4). When corrected for creatinine, speciated arsenic in urine was correlated with arsenic in house dust (*p* = 0.030). Age (*p* = 0.003) and body weight (*p* = 0.029) showed a significant positive association with speciated urinary arsenic levels but were negatively associated with speciated arsenic levels corrected for creatinine (Table 4). The only significant associations between urinary arsenic and categorical exposure variables were visiting a local orchard (*p* = 0.002) or a home undergoing renovation (*p* = 0.027) within a week of sampling (Table 5).

Age-adjusted regression models failed to indicate an association between arsenic in urine and the environmental variables. Increasing the considered age range to younger than 13 years of age (*n* = 76 for soil; *n* = 88 for house dust concentration) or to all ages (*n* = 249 for soil; *n* = 278 for house dust concentration) did not result in a significant association between speciated urinary arsenic and the environmental variables in age-adjusted regression models. Because a “base” model could not be established, further multiple regression models were not run.

Results for children younger than 13 years of age were generally similar to those in children younger than 7 years: for example, highest correlation between speciated urinary arsenic levels and mean soil arsenic level (*r* = 0.201, *p* = 0.081) and significant association of speciated urinary arsenic with age (*r* = 0.294, *p* = 0.001). Creatinine-corrected speciated urinary arsenic, however, was not significantly correlated with arsenic in house dust, and urinary arsenic associations with body weight or visiting a house with renovations were not significant.

For all participants, speciated urinary arsenic levels had the highest correlation with arsenic concentration in house dust (*r* = 0.110, *p* = 0.068), were negatively correlated with eating homegrown produce (*r* = −0.097, *p* = 0.043), and were higher for those who ate rice or rice products [GM (*n*) = 4.5 μg/L (127) vs. 3.7 μg/L (308); *p* = 0.003]. Age was negatively correlated with speciated (*r* = −0.158, *p* < 0.001) urinary arsenic levels, and males had slightly higher speciated urinary arsenic levels (GM = 4.17 μg/L vs. 3.63 μg/L; *p* = 0.029).

## Discussion

### Comparison with other sites.

The ATSDR (2000) reported a reference level of 50 μg/L for total arsenic in urine, but not for speciated arsenic, the better measure of exposure to inorganic arsenic. Toenail arsenic levels were below the reported reference level of 1 mg/kg (ATSDR 2000).

Speciated urinary arsenic levels of young children (i.e., < 7 years of age) in Middleport were low compared with levels reported for children at other sites with higher soil arsenic levels (Table 6). Results of Polissar et al. (1987) reflected high levels of arsenic emitted from a recently operating smelter. Urinary arsenic levels for children were also much higher than for adults, unlike what we found at Middleport. After smelter closure, urinary results were considerably lower [Tacoma-Pierce County Health Department (TPCHD) 1988; Table 6].

Middleport urinary arsenic levels for all ages combined were also consistent with results reported for “control” populations including all ages (Hinwood et al. 2003b, 2004; Polissar et al. 1987, 1990).

### Biomarker-based measures of arsenic exposure.

Because inorganic arsenic also occurs naturally in food and water (ATSDR 2000; Schoof et al. 1999; Yost et al. 2004), low levels of speciated arsenic are expected in urine. Although organic arsenic in seafood and some terrestrial organisms (Irgolic et al. 1999) primarily affects total rather than speciated arsenic in urine, other forms of arsenic in seafood (e.g., arsenosugars in bivalves and seaweed) can contribute to methylated arsenic species in urine (Le et al. 1999; Polissar et al. 1990).

Arsenic in urine is considered the most reliable biomarker of recent arsenic exposure (e.g., a few days to a week; ATSDR 2000). Biomonitoring of communities typically uses first-morning-void samples because 24-hr urine collection particularly from children is inconvenient and missed samples are likely (Hwang et al. 1997b). Hwang et al. (1997a, 1997b) analyzed two consecutive, first-morning-void urine samples for approximately 300 children and 24-hr urine in a subset of 25 children, but used the first-morning-void samples in the exposure analysis, and reported no differences in study results between using the average or highest of the two first-morning-void samples.

Toenail and hair samples reflect longer term exposure but are not easily related to a daily dose and are confounded by external arsenic contamination that is not easily removed (Harkins and Susten 2003; Hindmarsh et al. 1999; Hinwood et al. 2003a).

### Sources and factors potentially affecting arsenic exposure.

Several elements of the study increased the likelihood of detecting exposures from arsenic in soil: *a*) the study focused on the age group with greatest soil exposure; *b*) approximately half of young children in the community participated; *c*) biomonitoring occurred during summer when soil exposures would be highest; *d*) urinary samples were analyzed for the specific forms of arsenic related to inorganic arsenic exposure; and *e*) the study design evaluated the statistical relationship between environmental samples and individual urinary arsenic levels, including evaluation of other factors affecting exposure, rather than simply comparing mean urinary arsenic levels with another community.

As also noted by a study in Bingham Creek, Utah, [University of Cincinnati Department of Environmental Health (UCDEH) 1997], increased awareness had little effect on exposure. Few parents attempted to limit their children’s exposure to soil (5 of 76 for < 7 years of age; 8 of 135 for < 13 years of age), and urinary arsenic levels were not significantly lower.

### Correlations between environmental arsenic and urinary arsenic levels.

Lack of correlation between urinary arsenic and environmental measures may be the result of low arsenic levels in Middleport or limited sample size (participation in soil sampling was likely limited by the site agreement that data be shared with the state environmental agency) relative to the weakness of the correlations. Based on the highest estimated correlation coefficient between speciated urinary arsenic and soil, the sample size of children would have to be larger (≥ 203) than the estimated population of young children (164) to detect a significant correlation at α = 0.05. Speciated urinary arsenic, however, was not correlated with arsenic in soil in Bingham Creek, which involved 696 children (UCDEH 1997).

Reported correlations between speciated or inorganic urinary arsenic and measures of arsenic in soil are weak (*r* = 0.12–0.25, Hwang et al. 1997a, 1997b; Spearman *r* = 0.39, Hinwood et al. 2004). An increase in soil arsenic from 10 to 100 mg/kg would increase the GM of speciated urinary arsenic in young children in Middleport by only 1.2 times, according to Hwang et al. (1997a). Lower bioavailability and ingestion rates of arsenic in soil relative to food and water, combined with relatively low soil arsenic concentrations, are likely factors in the low soil arsenic exposure in this community.

Creatinine adjustment of urinary arsenic did not improve correlations between urine and soil arsenic levels, although a correlation with house dust became significant. Larger studies reported similar findings, except that urinary arsenic was not correlated with house dust at one study location (Anaconda, MT; Hwang et al. 1997a, 1997b) and only weakly correlated (*r* = 0.08; *p* < 0.05) in Bingham Creek (UCDEH 1997). Because creatinine excretion levels vary with muscle mass, sex, age, diet, genetic factors, diseases, and time, creatinine is not an accurate measure of sample dilution (Barr et al. 2005). Collection at a standard time (first morning void) and using 2-day composite samples likely reduced sample dilution variation in our study.

Although quantifying environmental exposure for individuals is uncertain, young children are more likely to be exposed to their immediate home environment, and composite soil samples are more representative of exposure over a yard than are a few discrete point samples (Hwang et al. 1997a; UCDEH 1997).

For several participants, collection of house dust samples, particularly for measures of concentration, was delayed by scheduling difficulties. Such delays are not expected to affect the arsenic concentration in house dust as much as for arsenic loading, unless a source of arsenic has increased (e.g., burning treated wood). Arsenic loading thus may be less representative of conditions at the time of urinary sampling.

### Indirect indicators of potential arsenic exposure.

Unlike the direct correlations with soil data, these indirect analyses (survey responses, geographic distribution of urine data) included data from nearly all 77 young children. Higher urinary arsenic levels in the few children who visited orchards may reflect exposure from historical use of arsenic-containing pesticides. Consumption of garden vegetables has not been associated with increased urinary arsenic levels at other sites, as well (Hwang et al. 1997a; Polissar et al. 1987; UCDEH 1997).

Rice consumption may increase arsenic exposure, as observed in the total study population, because compared with other foods, a large percentage of arsenic in rice is in the inorganic form (Schoof et al. 1999). Thus, although we were not able to detect increased exposure from arsenic in soil, we may have been able to detect small contributions from dietary inorganic arsenic, a primary source of inorganic arsenic exposure (Meacher et al. 2002).

## Conclusions

The results of this study are consistent with studies involving larger populations and higher soil arsenic concentrations. Although our results may seem inconsistent with those of risk assessment, biomonitoring and risk assessment differ in their focus. Speciated arsenic in urine includes all sources of inorganic arsenic (e.g., diet and water). Consequently, measurement of increased soil exposure is limited by the magnitude of this exposure relative to background sources of inorganic arsenic. Risk assessments of soil incorporate health-protective policy to avoid underestimation of soil exposure, regardless of whether background exposures from other sources are higher. Explaining these differences to the community is important for communicating risks.

## Figures and Tables

**Table 1:** Demographic characteristics of the study area and study participants [*n* (%)].

	Study area	2000 U.S. Census^a^	Study participants
Total persons	1,930	1,917	439
Population by sex			
Male	874 (45)	908 (47)	206 (47)
Female	981 (51)	1,009 (53)	233 (53)
Unknown	75 (4)	—	—
Population by age (years)			
< 5	104 (5)	141 (7)	43 (10)
< 7 (i.e., younger than 84 months)	164 (8)	—	77 (18)
5–9	116 (6)	129 (7)	70 (16)
10–14	105 (5)	172 (9)	42 (10)
15–19	128 (7)	155 (8)	28 (6)
≥ 20	997 (52)	1,320 (69)	256 (58)
Unknown	465 (25)	—	—
Individuals by race (%)			
White	—	1,867 (97)	402 (92)
African American	—	16 (< 1)	9 (2)
Native American	—	5 (< 1)	13 (3)
Asian	—	9 (< 1)	0
Other	—	20 (1)	8 (< 2)
Unknown	—	15 (< 1)	7 (< 2)
Total households	826	757	167
With children younger than 7 years	106 (13)	—	55 (33)
With children younger than 13 years	161 (19)	—	75 (47)
With individuals younger than 18 years	227 (27)	286 (38)	90 (54)
Income ≤ $40,000/year	—	358 (47)^b^	72 (43)
Income > $40,000/year	—	399 (53)^b^	82 (49)
Unknown	—	—	13 (8)

a Within Middleport village boundaries.

b 2000 U.S. Census income categories (U.S. Census Bureau 2000) were less than or greater than $35,000.

**Table 2:** Summary of arsenic concentration (μg/L) in urine.

			Individual arsenic species		
	Total arsenic	Speciated arsenic	Inorganic arsenic	MMA	DMA
Children < 7 years (*n* = 77)					
GM (GSD)	15.1 (1.8)	4.0 (2.2)	0.81 (1.5)	0.54 (1.9)	2.5 (2.9)
Range	2.1–59.6	0.89–17.7	0.31–2.1	0.12–2.1	0.27–13.8
Children < 13 years (*n* = 142)					
GM (GSD)	15.7 (1.7)	4.6 (2.1)	0.83 (1.4)	0.55 (1.8)	3.0 (2.6)
Range	2.1–59.9	0.89–19.9	0.31–2.7	0.11–2.4	0.27–17.1
Children ≥ 7 years/adults (*n* = 362)					
GM (GSD)	15.8 (2.1)	3.8 (1.9)	0.78 (1.4)	0.44 (1.8)	2.5 (2.3)
Range	3.9–773	0.91–19.9	0.31–2.7	0.024–2.4	0.17–17.1
All participants (*n* = 439)					
GM (GSD)	15.7 (2.0)	3.9 (1.9)	0.78 (1.4)	0.46 (1.8)	2.5 (2.4)
Range	2.1–773	0.89–19.9	0.31–2.7	0.024–2.4	0.17–17.1

**Table 3:** Summary of arsenic concentration in soil and house dust.

	Soil (mg/kg)		House dust	
	Property average	Property maximum	Arsenic concentration (mg/kg dust)	Surface loading of arsenic (μg/100 cm^2^)
All households				
No. of homes sampled	85	85	96	111
GM (GSD)	20.6 (2.0)	24.7 (2.2)	10.8 (3.0)	0.071 (4.4)
Range	4.6–340	6.2–1,124	1.0–172	0.004–2.97
Households with children younger than 7 years				
No. of homes sampled	29	29	36	37
GM (GSD)	19.9 (1.6)	23.8 (1.7)	11.2 (3.1)	0.058 (4.0)
Range	10.4–46.4	10.4–58.8	1.7–172	0.004–0.77

**Table 4:** Correlation of urinary arsenic levels with environmental arsenic levels and numerical exposure factors for children younger than 7 years of age.

					Correlation with urinary arsenic (μg/L)	
Exposure factor	No.	Mean ± SD	Median	Range	Speciated arsenic	Creatinine- corrected speciated arsenic
Soil arsenic average (mg/kg)	41	18.8 (1.6)^a^	15.6	10.4–46.4	0.137	−0.019
Soil arsenic maximum (mg/kg)	41	22.9 (1.7)^a^	22.6	10.4–58.8	0.045	−0.132
House dust arsenic concentration (mg/kg)	52	10.6 (2.9)^a^	9.5	1.7–172	0.049	0.301*
House dust surface loading (μg As/100 cm^2^)	53	0.058 (4.1)^a^	0.056	0.004–0.77	0.090	0.232
Age of child (years)	77	4.3 ± 2	4.7	0.1–7	0.331**	−0.263*
Weight (kg)	75	18.3 ± 6.4	18	5–35	0.253*	−0.317**
Time playing in outdoor area (days/week)	70	5.2 ± 1.7	5	1–7	−0.150	0.003
Washed hands (times/day)	77	4.4 ± 3.1	3	0–20	−0.052	−0.275*
Playing near creeks (days/week)	10	4.0 ± 2.5	4	1–7	0.160	0.152
Playing in orchards (days/week)	3	1.7 ± 0.6	2	1–2	−0.484	−0.868

Urinary and environmental arsenic variables were log transformed before analysis. Other numerical survey variables not shown did not have significant correlations: body mass index, number in household, and frequency of bathing, taking food/drink outdoors, drinking tap water, and eating homegrown produce, seafood, and rice products.

a GM (GSD).

* *p* < 0.05.

** *p* < 0.01.

**Table 5:** Summary of categorical questionnaire variables and associated urinary arsenic levels (μg/L) for children younger than 7 years of age.

	Response	No.	Speciated arsenic GM (GSD)
Sex	Female	40	3.80 (2.46)
	Male	37	4.25 (2.00)
Visited a house/building with ongoing renovations?	Yes	6	7.93 (1.62)*
	No	68	3.76 (2.21)
	Don’t know	1	5.75 (—)
Limit child’s exposure to soil or dust?	Yes	5	2.18 (2.76)
	No	71	4.12 (2.17)
Play near creeks?	Yes	10	4.23 (2.46)
	No	67	3.98 (2.22)
Spent time at local orchard or produce farm?	Yes	3	5.43 (1.04)*
	No	73	3.96 (2.28)

* Significant difference in urinary arsenic levels between “yes” and “no” responses (*t*-test; *p* < 0.05). Other categorical responses with no significant differences: type of ground play surface, playing with outdoor pet, age of house, frequency of sucking fingers, frequency of putting objects in mouth, family income, exposure to smoking, daycare attendance, race, pacifier use, herbal medicine use, exposure to treated wood, street paved, eaten homegrown produce, eaten seafood, eaten rice/rice products, large digging or moving soil projects in last year. No significant results for creatinine-corrected speciated arsenic.

**Table 6:** Speciated urinary arsenic and soil arsenic levels for young children at various sites.

	Speciated urinary arsenic concentration (μg/L)			Soil arsenic concentration (mg/kg)		
	*n*	GM (GSD)	Range	*n*	GM (GSD)	Range
Middleport, NY, 2003	77	4.0 (2.2) 5.3 ± 3.0^a^	0.89–17.7	29	19.9 (1.6) 22.5 ± 11.7^a^	10.4–58.8
Bingham Creek, UT (UCDEH 1997)						
Residences near Bingham Creek channel	696	5.86 (1.96)	ND–35	1,045	27 (1.8)	4–623
Ruston/North Tacoma, WA, 1985–1986 (Polissar et al. 1987)						
< 0.5 miles from smelter	118	52.1 (42.5)^b^	NR	45	352 (410)^b^	12–2,069
0.5–1.2 miles from smelter	97	22.5 (29.3)^b^	NR	40	125 (109)^b^	9–1,322
1.5–8.5 miles from smelter	49	13.7 (10.3)^b^	NR	34	29.6 (49)^b^	2–290
Reference site (Bellingham, WA)	4	13.3 (3.3)^b^	NR	10	6.6 (2.7)^b^	2–10
> 100 miles from smelter						
Ruston/North Tacoma, WA, 1987 (TPCHD 1988)						
< 0.5 miles from smelter	88	16.2 (16)	NR	NR	NR	NR
Anaconda, MT (Hwang et al. 1997a, 1997b)						
Close to smelter	177	9.5 (1.7)	NR–16.4	876	286^c^	NR
Intermediate	62	7.5 (1.5)	NR–19.0	405	150^c^	NR
Remote	42	7.1 (1.8)	NR–12.1	302	90^c^	NR

Abbreviations: ND, not detected; NR, not reported.

a Arithmetic average ± SD.

b Arithmetic averages were reported for urine and soil. Urine values are the weighted arithmetic average from separate results for male and female.

c Average yard soil arsenic concentrations for Anaconda are the GM calculated as the weighted average of all soil samples.
